# Anorexia nervosa-associated pancytopenia mimicking idiopathic aplastic anemia: a case report

**DOI:** 10.1186/s12888-018-1743-6

**Published:** 2018-05-25

**Authors:** Masahiro Takeshima, Hiroyasu Ishikawa, Akihiro Kitadate, Ryo Sasaki, Takahiro Kobayashi, Hiroshi Nanjyo, Takashi Kanbayashi, Tetsuo Shimizu

**Affiliations:** 1 Department of Neuropsychiatry, Akita University Graduate School of Medicine, 1-1-1,Hondo, Akita City, Akita 010-8543 Japan; 2 Department of Neuropsychiatry, Nakadori Rehabilitation Hospital, 6-1-58, Nakadori, Akita City, Akita 010-0001 Japan; 3 Division of Hematology/Oncology, Department of Medicine, Kameda General Hospital, 929 Higashi-chou, Kamogawa City, Chiba 296-8602 Japan; 4 Department of Neuropsychiatry, Akita City Hospital, 4-30, Matsuoka-machi, Kawamoto, Akita City, Akita 010-0933 Japan; 5 Department of Hematology, Nephrology and Rheumatology, Akita University Graduate School of Medicine, 1-1-1, Hondo, Akita City, Akita 010-8543 Japan; 6 Division of Clinical Pathology, Akita University Graduate School of Medicine, 1-1-1, Hondo, Akita City, Akita 010-8543 Japan

**Keywords:** Anorexia nervosa, Aplastic anemia, Bone marrow, Gelatinous transformation, Pancytopenia

## Abstract

**Background:**

Patients with anorexia nervosa (AN) often present with pancytopenia. In most cases described in the literature, AN with pancytopenia demonstrates gelatinous marrow transformation (GMT), which is a typical bone marrow feature of malnutrition. Differentiation of AN-associated pancytopenia from other types of pancytopenia, especially idiopathic aplastic anemia (IAA), has not been studied. We encountered a case of pancytopenia in a patient with AN and relatively poor nutritional status, whose hematological findings mimicked those of IAA, specifically fatty bone marrow and absence of GMT.

**Case presentation:**

The patient was a 32-year-old woman with poorly controlled AN. At 31 years of age, her body mass index (BMI) had fallen from 17.0 kg/m^2^ to below 13.8 kg/m^2^. The patient presented with ongoing fatigue and thus was examined by a hematologist. Hematological findings were consistent with IAA: peripheral blood tests revealed pancytopenia, whereas the bone marrow displayed fatty replacement without GMT. Despite the absence of bone marrow features typically seen in malnutrition, the patient’s hematological abnormalities had manifested after a decrease in body weight. Thus, although the bone marrow findings indicated IAA, we considered that the nutritional etiology of pancytopenia could not be thoroughly ruled out. Using nutritional therapy alone, the hematological abnormalities improved as BMI increased to 16.5 kg/m^2^. The final diagnosis was pancytopenia secondary to malnutrition because pancytopenia and fatty bone marrow improved after implementation of nutritional therapy alone.

**Conclusions:**

The present case is the first documented case of AN with pancytopenia for which bone marrow examination confirmed fatty marrow without any evidence of GMT. IAA and pancytopenia secondary to malnutrition can present the same clinical findings. This case is significant because it suggests a need to differentiate between malnutrition and IAA.

## Background

Anorexia nervosa (AN) is associated with various hematological abnormalities, with approximately 3% of cases exhibiting pancytopenia [1]. In almost all cases of AN with pancytopenia reported thus far, bone marrow analyses show atrophy of fat cells and loss of hematopoietic cells, with deposition of an amorphous gelatinous material [2–10]. This phenomenon has been described as gelatinous marrow transformation (GMT) [11, 12]. GMT is often observed in patients with pancytopenia due to malnutrition. However, to the best of our knowledge, the differentiation between malnutrition-associated etiology and other etiologies of pancytopenia in AN was not discussed in previous reports describing patients with AN. We herein report a case of pancytopenia in a patient with poor nutritional status, whose peripheral blood and bone marrow findings indicated idiopathic aplastic anemia (IAA).

## Case presentation

A 32-year-old Japanese woman with AN and pancytopenia was admitted to the psychiatric department of our hospital. The patient had no other remarkable medical or familial history. There was no occupational history indicating exposure to organic solvents (e.g., benzene).

The patient started binge eating and purging at 14 years of age. At 16 years of age, she was diagnosed with AN, and had multiple hospitalizations in this regard. The patient’s first admission to our department was at 26 years of age, at which time her body mass index (BMI) was 9.5 kg/m^2^ (weight, 22 kg; height, 152 cm). The patient had mild, transient bicytopenia with a low white blood cell (WBC) count (3000 cells/μL; reference range, 4000–9000 cells/μL) and a low hemoglobin (Hb) level (10.3 g/dL; reference range, 12.0–15.2 g/dL). These abnormalities improved with nutritional therapy. At the time of discharge, the patient’s weight had improved, with a BMI of approximately 17 kg/m^2^.

At 32 years of age, the patient’s binge eating and purging behavior worsened again, and she began to lose weight. Five months prior to her eventual hospitalization (BMI, 15.0 kg/m^2^), no hematological abnormalities were identified. At 46 days prior to the hospitalization, because of ongoing fatigue, she was examined by a hematologist at our hospital. The patient was determined to be underweight, with a body weight of 31.9 kg and a BMI of 13.8 kg/m^2^. Peripheral blood analysis confirmed pancytopenia with the following findings: low WBC count, 2500 cells/μL; low neutrophil count, 1010 cells/μL (reference range: 1600–5400 cells/μL); low eosinophil count, 0 cells/μL (reference range: 80–610 cells/μL); low basophil count, 30 cells/μL (reference range: 0–180 cells/μL); normal lymphocyte count, 1280 cells/μL (reference range: 1060–4190 cells/μL); normal monocyte count, 200 cells/μL (reference range: 90–690 cells/μL); low reticulocyte count, 14.4 × 10^3^ cells/μL (reference range: 35.0–125.0 × 10^3^ cells/μL); low Hb level, 6.1 g/dL; high mean corpuscular volume, 113.9 fL (reference range: 80.0–100.0 fL); and low platelet (Plt) count, 10.6 × 10^4^ cells/dL (reference range: 11.7–32.9 × 10^4^ cells/dL). Blood biochemistry studies showed low serum levels of folate (3.0 ng/mL; reference range: 4.0–19.9 ng/mL) but normal serum levels of iron and vitamin B12. The etiology of pancytopenia was tentatively suspected to be folate deficiency, and thus the patient was prescribed folate at 10 mg/day. At 28 days prior to hospitalization, the folate level had recovered within normal limits, but the peripheral blood counts had worsened (low WBC count, 2200 cells/μL; low Hb level, 2.5 g/dL; and low Plt count, 8.6 × 10^4^ cells/dL).

The patient was temporarily admitted to the hematology department of our hospital for blood transfusions. Laboratory tests yielded negative results for autoimmune disorders (including systemic lupus erythematosus), malignant lymphoma, infectious diseases, hemophagocytic lymphohistiocytosis, and hypersplenism. The patient was offered nutritional therapy to improve her weight but she refused and was therefore discharged following blood transfusions. After discharge, outpatient follow-up was performed by the hematology department. However, the patient’s hematologic condition did not improve. Despite receiving blood transfusions twice monthly, the patient was eventually hospitalized once again for severe anemia. The patient believed that her hematological problems were not related with her eating behavior and persistently refused nutritional therapy. Finally, she was admitted to the psychiatric department to receive both hematologic and psychiatric support.

On admission, the patient’s body weight was 30 kg and BMI was 13.0 kg/m^2^. The vital signs were normal. Physical examination revealed no abnormalities other than bilateral non-pitting edema of the lower legs. The patient received folate (10 mg/day), sertraline (50 mg/day), zolpidem (10 mg/day), and flunitrazepam (2 mg/day). Toxicology analysis was not performed because the patient presented no feature indicating exposure to toxic compounds. Laboratory findings were as follows: low WBC count, 2100 cells/μL; low reticulocyte count, 9.4 × 10^3^ cells/μL; low Hb level, 5.7 g/dL; and low Plt count, 4.1 × 10^4^ cells/dL. The differential WBC count was normal. Serum levels of iron, folate, vitamin B12, zinc, copper, and ceruloplasmin were within normal ranges. These results did not identify the etiology of the hematological problems. The patient persistently refused nutritional therapy and hoped that some other causes of pancytopenia (other than malnutrition) would be found. On hospitalization day 7, upon obtaining written informed consent from the patient, we performed bone marrow aspiration and biopsy.

**Fig. 1:**
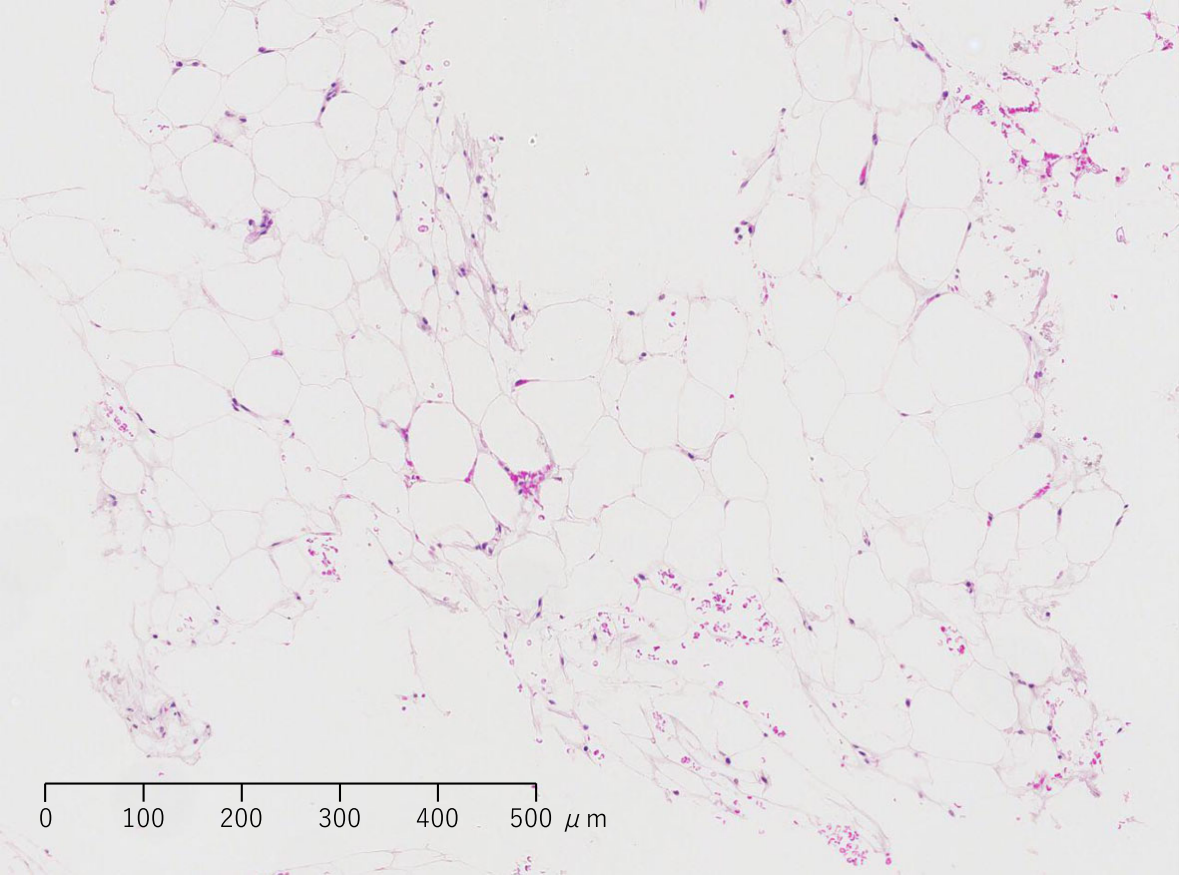
Bone marrow biopsy of a patient with poorly controlled anorexia nervosa and severe hematological abnormalities. On hospitalization day 7, hematoxylin-eosin staining of the bone marrow sample revealed hematopoietic hypoplasia with increased fatty material but no apparent gelatinous material

Bone marrow aspiration revealed low counts of nucleated cells (5.5 × 10^3^ cells/μL; reference range: 100–200 × 10^3^ cells/μL) and megakaryocytes (< 15.6 cells/μL; reference range: 50–150 cells/μL). Bone marrow biopsy revealed hypoplasia but no dysplasia and confirmed the presence of fatty replacement with no gelatinous material (Fig. 1). It was notable that the bone marrow displayed fatty marrow findings without any evidence of GMT, which would generally indicate IAA. Moreover, no chromosomal abnormalities or cell surface abnormalities were found upon bone marrow examination. T1-weighted images from thoracolumbar magnetic resonance imaging confirmed homogenous high signal intensity, characteristic of fatty marrow. Detailed examination of peripheral blood and bone marrow findings indicated IAA. However, despite the absence of bone marrow features typically seen in malnutrition, the patient’s hematological abnormalities manifested after a decrease in body weight. Thus, although the biopsy findings indicated IAA, we considered that the nutritional etiology of pancytopenia could not be thoroughly ruled out.

After bone marrow aspiration and biopsy, the patient accepted nutritional therapy. At the time of admission, she was given 600 kcal/day intravenously. Energy intake was gradually increased, and the patient received oral nutrition amounting to 3200 kcal/day from hospitalization day 14 until discharge. The patient’s caloric intake was strictly managed, which resulted in weight restoration (Table 1). The hematological abnormalities began to improve with the gradual increase in caloric intake. By hospitalization day 11, the patient’s condition had improved to the point where she no longer required transfusions. On hospitalization day 28, the patient’s weight was 38.1 kg, and her BMI was 16.5 kg/m^2^. Pancytopenia had also improved (Table 1), and thus the patient was discharged the following day. Bone marrow aspiration and biopsy performed 2 weeks after discharge yielded normal findings. Because both blood and bone marrow findings improved after implementation of nutritional therapy alone, the final diagnosis was pancytopenia secondary to malnutrition.

**Table 1:** Laboratory data prior to and throughout the course of hospitalization in the present case

Day	−46	−28^a^	−25^b^	0^c^	+ 7	+ 14	+ 21	+28^d^	Unit	Normal range
WBC count	**2500**	**2200**	**2400**	**2100**	**1200**	**2400**	**3000**	4200	cells/μL	4000–9000
Hb	**6.1**	**2.5**	**7.1** ^**e**^	**5.7** ^**e**^	**4.2**	**9.6** ^**e**^	**8.3**	**8.3**	g/dL	12.0–15.2
MCV	113.9	122.0	95.8	92.4	95.7	95.2	96.7	98.5	fL	80.0–100.0
Reticulocyte count	**14.4**	N.A.	**13.2**	**9.4**	**24.3**	52.4	69.4	115.1	×10^3^ cells/μL	35.0–125.0
Plt count	**10.6**	**8.6**	**8.5**	**4.1**	**7.5**	**11.1**	14.5	22.6	×10^4^ cells/dL	11.7–32.9
BMI	13.8	12.9	N.A.	13.0	13.7	15.0	15.8	16.5	kg/m^2^	18.5–24.9

The hematological abnormalities improved by nutritional therapy alone

Bold font indicates abnormally low values

*WBC* white blood cell, *Hb* hemoglobin, *MCV* mean corpuscular volume, *Plt* platelet, *BMI* body mass index, *N.A* not available

^a^Admission to the hematology department

^b^Discharge from the hematology department

^c^Admission to the psychiatric department

^d^Discharge from the psychiatric department

^e^Value measured within a week of last transfusion

## Discussion and conclusions

To the best of our knowledge, no other literature reports have described patients with AN who present with peripheral blood and bone marrow findings consistent with IAA. In the present case, pancytopenia is believed to have been caused by malnutrition. Thus, the present case is the first documented case of AN with pancytopenia for which bone marrow examination confirmed fatty marrow without GMT. Therefore, it was necessary to differentiate the actual cause of pancytopenia from IAA, as analysis of peripheral blood and bone marrow led to the suspicion of IAA [13]. However, because blood and bone marrow findings improved after implementation of nutritional therapy alone, the final diagnosis was revised to pancytopenia secondary to malnutrition. These findings are significant because they suggest a potential need to differentiate between malnutrition and IAA.

Bone marrow pathology findings were also distinctive in the present case. Although cytopenia was severe, bone marrow examination did not indicate GMT, which is thought to be the most critical bone marrow finding among patients with eating disorders. A significant correlation has been reported among BMI, WBC count, and red blood cell count in patients with eating disorders, as well as between weight loss and the extent of bone marrow abnormalities [14]. Therefore, it was difficult to establish our patient’s diagnosis, as there was discrepancy between the results of bone marrow and blood tests.

Nearly all reported cases of eating disorders accompanied by pancytopenia included findings of gelatinous bone material [2–10] (Table 2). Upon examining patients with eating disorders, Abella et al. [14] classified bone marrow findings into four degrees of severity: normal, hypoplastic or aplastic without gelatinous degeneration, hypoplastic or aplastic with partial or focal gelatinous degeneration, and hypoplastic or aplastic with complete gelatinous degeneration of the bone marrow. Although the patients described by Abella et al. did not have pancytopenia, it is notable that those with hypoplastic or aplastic marrow without gelatinous degeneration exhibited increased bone marrow fat fraction owing to increased adipocyte diameter [14]. In the present case, bone marrow findings were consistent with a hypoplastic or aplastic disorder (without gelatinous degeneration), as defined by Abella et al. [14]. Therefore, in such cases, abnormal bone marrow findings related to malnutrition cannot be differentiated based on the presence of fatty marrow, which is a typical bone marrow feature of IAA. Caution is therefore necessary, to avoid the potential pitfalls of a diagnosis based on hematologic parameters alone.

**Table 2:** Summary of bone marrow findings in previously reported patients with anorexia nervosa and pancytopenia

Age	Sex	BMI (kg/m^2^)	Hematopoietic cells in the bone marrow	Gelatinous material in the bone marrow	Fat cells in the bone marrow	Year of publication	Reference
32	F	13	hypocellular	(−)	increase	2018	present case
16	F	ND	hypocellular	(+)	decrease	2016	[7]
33	F	12.3	hypocellular	(+)	decrease	2013	[4]
18	F	12.1	hypocellular	(+)	lack	2013	[3]
28	M	18.2	hypocellular	(+)	increase	2004	[9]
28	F	12.3	hypocellular	(+)	lack	2003	[6]
20	F	10.7	hypocellular	(+)	decrease	1998	[10]
17	F	11.4	hypocellular	(−)	lack	1993	[15]
36	F	10.2	hypocellular	(+)	lack	1987	[8]
17	ND	ND	hypocellular	(+)	lack	1981	[5]
62	ND	ND	hypocellular	(+)	lack	1981	[5]
14	ND	ND	hypocellular	(+)	decrease	1981	[5]
18	F	ND	hypocellular	(+)	decrease	1979	[1]

In most cases described in the literature, anorexia nervosa with pancytopenia demonstrated gelatinous marrow transformation

*BMI* body mass index, *F* female, *M* male, *ND* not described

An accurate differential diagnosis of pancytopenia is crucial for selection of the most appropriate treatment. Should a case like the one described here be misdiagnosed as IAA, standard treatments such as immunotherapy and bone marrow transplant may be implemented, which would place an unnecessary burden on the patient. In cases where it is difficult to distinguish between IAA and pancytopenia due to malnutrition, we recommend a course of treatment focused on nutritional therapy, as well as careful follow-up.

## Abbreviations

AN: Anorexia nervosa
BMI: Body mass index
GMT: Gelatinous marrow transformation
Hb: Hemoglobin
IAA: Idiopathic aplastic anemia
Plt: Platelet
WBC: White blood cell
